# Meat intake among adults: a population-based study in the city of Campinas, Brazil. A cross-sectional study: Ingestão de carnes em adultos: estudo de base populacional na cidade de Campinas, Brasil. Um estudo transversal

**DOI:** 10.1590/1516-3180.2015.01691609

**Published:** 2016-03-18

**Authors:** Jaqueline Contrera Avila, Verônica Gronau Luz, Daniela de Assumpção, Regina Mara Fisberg, Marilisa Berti de Azevedo Barros

**Affiliations:** I RD. Registered Dietitian, School of Medical Sciences, Universidade Estadual de Campinas (Unicamp), Campinas, São Paulo, Brazil.; II RD, MSc, PhD. Assistant Professor, Universidade Federal da Grande Dourados, Dourados, Mato Grosso do Sul, Brazil.; III RD, MSc, PhD. Field coordinator, Nutrition survey of Campinas, School of Medical Sciences, Universidade Estadual de Campinas (Unicamp), Campinas, São Paulo, Brazil.; IV RD, MSc. PhD. Associate Professor, School of Public Health, Universidade de São Paulo (USP), São Paulo, São Paulo, Brazil.; V MD, MSc, PhD. Professor, School of Medical Sciences, Universidade Estadual de Campinas (Unicamp), São Paulo, Brazil.

**Keywords:** Meat, Health surveys, Adult, Eating, Diet

## Abstract

**CONTEXT AND OBJECTIVE:**

Meat is a food with high nutritional density that has significant participation in the Brazilian diet. However, in excess it can cause harm to health. The aim of this study was to analyze the meat intake (g/day) among adults according to sociodemographic, behavioral and health situation characteristics, and to assess the types of meat most consumed.

**DESIGN AND SETTING:**

Cross-sectional population-based study conducted in the city of Campinas, São Paulo, Brazil, in 2008 and 2009.

**METHODS:**

Two-stage cluster sampling was used. The analysis included 948 adults between 20 and 59 years, who were participants in the Campinas Health Survey. Meat intake was assessed using 24-hour dietary recall.

**RESULTS:**

The mean meat intake adjusted for sex and age was 182.3 g (95% CI: 170.6-193.9 g), with significantly lower intake among women, individuals aged 50 years or over, those with the presence of two or more self-reported chronic diseases and those with three or more health complaints. Higher meat intake was found in segments with intermediate monthly family income (between 1 and 3 minimum wages), those with 16 or more appliances per household and those who consumed soft drinks seven days a week. Beef was consumed most frequently (44%) among the meats in the diet, followed by poultry, fish and pork.

**CONCLUSION:**

The data from this study reveal high meat intake in the population of Campinas and identify the segments that need to be prioritized for strategies directed towards appropriate meat intake.

## RESUMO

**CONTEXTO E OBJETIVO:**

As carnes são alimentos com elevada densidade nutricional, apresentam expressiva participação na dieta dos brasileiros, porém em excesso provocam prejuízos à saúde. O objetivo deste estudo foi analisar a ingestão de carnes (g/dia) entre adultos segundo características sociodemográficas, comportamentais e de situação de saúde, além de avaliar os tipos de carnes mais consumidos.

**DESENHO E LOCAL:**

Estudo transversal de base populacional, realizado em Campinas, SP, Brasil, em 2008 e 2009.

**MÉTODOS:**

A amostra foi obtida por conglomerados e em dois estágios. Foram analisados 948 adultos (20-59 anos), participantes do Inquérito de Saúde de Campinas. O recordatório alimentar de 24 horas foi utilizado para estimar a ingestão de carnes.

**RESULTADOS:**

A ingestão média de carnes ajustada por sexo e idade foi de 182,3 g (IC 95%: 170,6-193,9 g), sendo significativamente menor nas mulheres, nos indivíduos com 50 anos ou mais, nos que relataram duas ou mais doenças crônicas e nos que apresentavam três ou mais queixas de saúde. Maior ingestão de carnes foi encontrada nos segmentos com renda familiar mensal intermediária entre 1 e 3 salários mínimos, com 16 ou mais equipamentos no domicílio, assim como aqueles que ingeriam refrigerantes nos 7 dias da semana. A carne bovina foi a mais frequente (44%) entre as carnes presentes na dieta, seguida de aves, processadas, peixes e suínas.

**CONCLUSÃO:**

Os dados desta pesquisa revelam elevada ingestão de carnes na população de Campinas e identificam os segmentos que devem ser priorizados para estratégias direcionadas a adequar a ingestão desse alimento.

## INTRODUCTION

Meat is a food group with significant participation in the Brazilian diet, and is used in the main course of most meals. It is a food with high nutrient density that provides an important source of high-quality proteins, vitamins and minerals for the Brazilian population, especially as a source of vitamin B12 and heme-iron.^1^

Brazil is also the second largest producer of beef in the world, and ranks highly with regard to production levels of other meats like chicken and pork.^2^^,^^3^

According to a national dietary survey, the Brazilian meat intake corresponds to 151.8 g/day.^4^ Compared with other foods, meat has greater participation than fruits (86.1 g/day), vegetables (24.6 g/day) and legumes (40.7 g/day), thus demonstrating its significant participation and intake.^4^ Other Brazilian surveys have also indicated that meat intake has increased over the years.^5^ Between the periods of 1974-1975 and 2002-2003, the participation of meat in the diet increased by almost 50%.^6^

The Brazilian Ministry of Health recommends that the maximum total meat intake should be 100 g per day, which corresponds to a portion of 190 kcal.^7^ The latest Brazilian dietary guidelines also emphasize that unprocessed lean meat should form part of a nutritionally adequate diet and it is recommended that the intake of red and processed meat should be reduced.^1^

The World Cancer Research Fund International (WCRF) has established a maximum recommendation of 500 g for red and processed meat per week,^8^ since these are the types of meat with higher quantities of cholesterol and saturated fat. These types of meat have been described in longitudinal analyses as risk factors for chronic diseases such as colorectal cancer,^8^ type 2 diabetes mellitus,^9^ atherosclerosis and other cardiovascular diseases.^10^^,^^11^

## OBJECTIVE

Given the importance of meat intake within the national scenario and the potential problems relating to excessive meat intake, the objective of this study was to describe the average meat intake (g/day) among adults ages 20-59 years old in the city of Campinas, São Paulo, Brazil, according to sociodemographic variables, health-related behavior, morbidities and body mass index (BMI); and also to identify the types of meat consumed by this population.

## METHODS

This was a cross-sectional population-based study developed using data from the Campinas Health Survey (ISACAMP 2008/2009), which obtained information from non-institutionalized individuals who were living in the urban area of the city of Campinas between February 2008 and April 2009.

The survey sample was determined through two-stage cluster sampling. In the first stage, 50 census tracts with probability proportional to size (number of households) were drawn. Considering the time that had elapsed since the census of 2000, addresses of selected tracts were updated. In the second stage, households were drawn.

The population was divided into three age domains: adolescents (10-19 years), adults (20-59 years) and elderly people (60 years or over). Independent samples of 1,000 people in each domain were drawn, taking into consideration the maximum variability of the frequencies of the events studied (P = 0.50), 95% confidence level, sampling error of between 4 and 5 percentage points and a design effect of 2. To obtain the desired sample size while taking into account the predicted non-response rate of 20%, 2,150, 700 and 3,900 households were drawn for interviews with adolescents, adults and elderly people, respectively. The estimated number of households was calculated based on the person/household ratio in each age domain. The interviews were conducted directly with residents within the age group drawn for that specific household. For this study, we used data on adults of both genders.

The information was collected by means of a questionnaire that was structured into 14 thematic blocks and had been tested in a pilot study. It was administered by trained and supervised interviewers. The thematic block relating to dietary habits included a food frequency questionnaire, self-reported weight and height and one 24-hour dietary recall, in which the respondents reported all the foods and beverages eaten the day before the interview. Interviews covering different days of the week and months of the year were collected.

The 24-hour dietary recall was quantified so as to convert homemade measurements to grams or milliliters, using information available from homemade measurement tables,^12^^,^^13^ food labels and customer service centers.

The data from the 24-hour dietary recall were entered into the Nutrition Data System for Research, 2007 version (NCC Food and Nutrient Database, University of Minnesota, Minneapolis, MN, USA).

### Study variables

The dependent variable was the mean meat intake (g/day).

The set of independent variables analyzed was the following.

Socioeconomic and demographic information: gender, age (in years), education level (in years of school attendance), per capita household income (in numbers of minimum wages) and number of appliances in the household.

Health-related behavior: weekly frequency of fruit, vegetable and soft-drink consumption; smoking and alcohol consumption.

Morbidities and body mass index (BMI): self-reported number of chronic diseases that had been diagnosed by a doctor (hypertension, diabetes, cancer, arthritis, osteoporosis, asthma, tendonitis and circulation problems) and number of health complaints among the ones included in the checklist (such as frequent migraines, back pain, allergies, etc.). The BMI was calculated using self-reported weight and height. Nutritional status was classified in accordance with the World Health Organization’s recommendation for adults:^14^ underweight BMI < 18.5 kg/m^2^, eutrophic BMI between 18.5 and 24.9 kg/m^2^, overweight BMI between 25.0 and 29.9 kg/m^2^ and obese BMI ≥ 30 kg/m^2^.

The average meat intake was estimated and differences between the means of the subgroups investigated were ascertained by means of simple and multiple linear regression, considering a 5% significance level for associations with the variables analyzed. The means were adjusted for age and sex.

Meat was classified according to animal origin and the type of processing, as follows: beef, poultry, pork, fish and processed meat, i.e. meat of any animal origin that had been subjected to industrial processing, so as to manufacture sausages, hamburgers, nuggets and other meat products. The relative participation of meats in the diet was calculated by dividing the total for each meat group (g) by the total meat in the diet (g). The mean intake of the separate types of meat was also calculated, using the following four categorizations: red and processed; poultry; fish; and pork.

The interviews were typed into the database using Epidata 3.1 (Epidata Assoc., Odense, Denmark) and statistical analyses were done using the survey module of the Stata 11.0 software (Stata Corp., College Station, USA), which enables analysis on data from complex samples.

The project ISACAMP 2008 was approved by the Research Ethics Committee of the School of Medical Sciences at the State University of Campinas under the protocol no. 079/2007.

## RESULTS

In the present study, 957 adults were interviewed. Among these, 9 individuals refused to participate in the 24-hour dietary recall, and therefore 948 adults were evaluated, including 43 who did not report eating meat on the day before the interview. Females accounted for 504 individuals and males for 444. The participants’ mean age was 37.5 years (95% CI: 36.6-38.3): 37.9 years for females (95% CI: 36.9-38.8) and 37.0 years for males (95% CI: 36.0-38.0).

The mean energy intake in the study was 2,013.27 kcal (95% CI: 1,934.95-2,091.39). The mean energy intake for males was 2,290.42 kcal (95% CI: 2,169.78-2,411.05) and for females, 1,750.08 kcal (95% CI: 1,669.80-1,830.36).

Considering the types of meat, the mean meat intake comprised 73.8 g (95% CI: 69.6-78.0) for red and processed meats; 97.6 g (95% CI: 86.9-108.4) for poultry; 69.7 g (95% CI: 52.7-86.6) for pork; and 86.6 g (95% CI: 62.5-110.7) for fish.

The most prevalent type of meat consumed was beef (41%), followed by poultry (22.8%), processed (16.8%), fish (8.4%) and pork (7.9%).

The daily mean total meat intake was 191 g (95% CI: 179.1-202.8), and the intake was significantly lower among women, and among individuals aged 50 years or over, compared with those between 20 and 29 years of age. Meat was more often consumed among individuals who reported per capita family incomes of between one and three minimum wages, and among those who had 16 or more appliances in the household (Table 1).

**Table 1.**
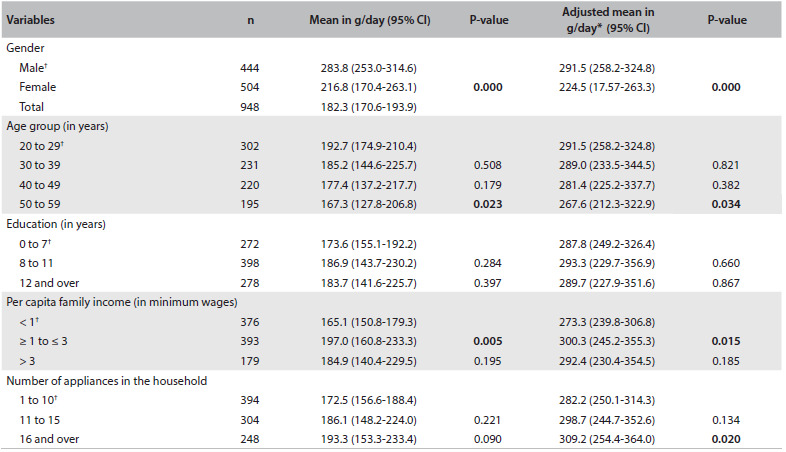
Mean meat intake (g/day) according to demographic and socioeconomic variables among adults between 20 and 59 years of age. Campinas Health Survey (ISACAMP, 2008/2009)

CI = confidence interval; ^*^adjusted for age and/or sex; ^†^reference category.

Table 2 shows that elevated meat consumption was associated with intake of soft drinks seven days a week, but that no other health-related behavioral patterns showed significant associations.

**Table 2.**
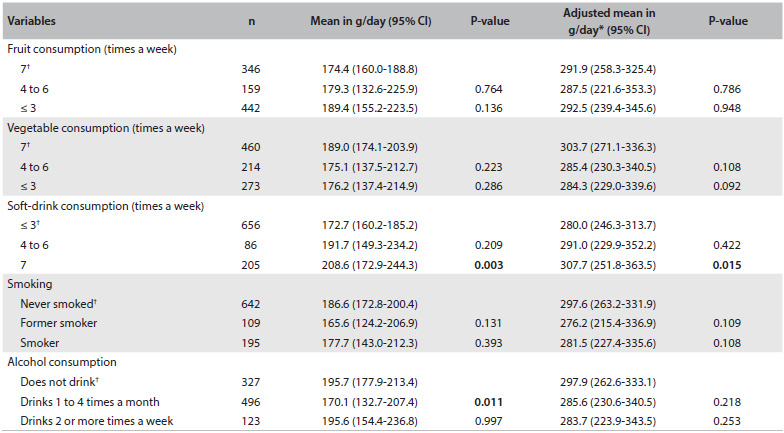
Mean meat intake (g/day) according to health-related behavior among adults between 20 and 59 years of age. Campinas Health Survey (ISACAMP, 2008/2009)

CI = confidence interval; *adjusted for age and sex; ^†^ reference category.

Table 3 shows that there was lower meat intake among individuals with two or more chronic diseases. Individuals who reported the presence of three or more health complaints also had statistically lower meat intake.

**Table 3.**
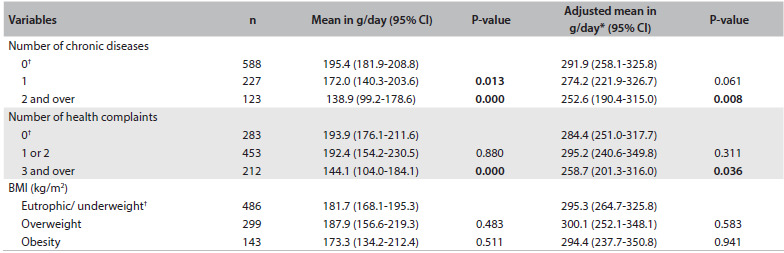
Mean meat intake (g/day) according to morbidities and body mass index among adults between 20 and 59 years of age. Campinas Health Survey (ISACAMP, 2008/2009)

CI = confidence interval; *adjusted for age and sex; ^†^reference category.

## DISCUSSION

The most important results from this study were that there was high dietary intake of meats among males and among individuals who reported monthly income of 1 to 3 minimum wages, those who had 16 or more appliances in the household and those who drank soft drinks on a daily basis. On the other hand, lower meat intake was observed among individuals aged 50-59 years than among those aged 20-29 years, and among individuals who reported the presence of two or more chronic diseases and those with three or more health complaints.

A separate analysis on the types of meat demonstrated that the highest intake was attributed to poultry, followed by red and processed meats. Higher demand for poultry has also been observed in the American population.^15^ The current literature does not demonstrate any higher incidence of colorectal cancer when the lean meat intake is within the recommended amounts.^16^^,^^17^ The American Heart Association recommends a maximum of 170 g of lean meat per day, which includes cooked poultry without skin and fish as important sources of high-quality protein in the diet.^18^

On the other hand, red and processed meats have been demonstrated to be risk factors for cardiovascular diseases^9^^,^^11^ and colorectal cancer.^16^^,^^17^ The findings relating to pork remain contradictory, since this meat has been shown to have no effect on metabolic syndrome,^19^ while its effect on colorectal cancer is unclear.^17^ At the same time, this meat type is usually analyzed inside the red meat subgroup,^9^ thus participating in the same group as beef and lamb in most analyses.

Furthermore, considering the distribution of the types of meat, there was greatest participation by beef, poultry and processed meats. Levy et al. worked on the Brazilian Household Budget Survey (BHBS) in 2008-2009 and observed that beef (4.42%), chicken (4.03%) and processed meat (2.22%) were the meats with highest participation in the national diet.^20^ Daniel et al. studied the American population and found similar distribution: 58% of the meat intake consisted of red meat (beef and pork), 32% poultry and 10% fish; processed meats were analyzed separately and corresponded to 22% of the overall meat intake.^21^

The average meat consumption of the population of Campinas (182.3 g) is higher than the national average. Evidence from the BHBS (2008/2009) showed that the national per capita meat intake was 151.8 g/day.^4^ Meat intake in Campinas was also greater than the total meat intake of 136.5 g found by Carvalho et al. in the city of São Paulo.^22^ Data from the National Health and Nutrition Examination Survey (NHANES) 2003 found that the average meat intake in the American population aged 20-49 years was 141 g/day, thus demonstrating that meat consumption in Campinas is high in comparison with national and international realities.^21^

Regarding gender differences, men were found to eat 67 g more meat than women. Other Brazilian studies have also indicated greater meat intake among men, especially for beef.^4^^,^^5^^,^^22^ Data from a telephone survey conducted in Brazil showed that men ate twice as much meat with visible fat as women did.^23^ This gender difference is related to the female concern for healthier food choices. Females are cautious about calories and fat content and usually select a diet with more fruits and vegetables instead of meat.^24^^,^^25^

Individuals aged 50 to 59 years had significantly lower meat intake than those aged 20-29 years, and this result was similar to what was found in NHANES.^21^ The Brazilian telephone survey of 2013 also revealed that meat consumption was lower among older individuals.^23^ Aging is accompanied by a greater risk of chronic diseases, which may influence individuals to improve their food choices and seek guidance from healthcare services, where disease control information is available.^26^

Regarding socioeconomic factors, higher dietary meat intake in the intermediate stratum of income was also observed by Carvalho et al. in the city of São Paulo.^22^ This pattern of meat intake among individuals with average income is associated with a trend towards eating meat as their income improved. The number of appliances in the household is considered to be a proxy variable for income, and it was observed that the individuals in the intermediate stratum of income were the ones with the greatest number of appliances in the household, thus explaining the greater meat intake in these categories.

Researchers using data from NHANES 2003 evaluated education levels as a proxy indicator for income, and demonstrated the same distribution of meat intake.^21^ This behavior indicates that the price of meat is still the determinant for access among lower-income populations.

Meat intake has shifted with the nutritional transition and the urbanization process, which has reduced the cost of meat based on government investment in livestock. This change has allowed lower-income individuals to purchase more meat, thereby bringing good protein and micronutrient sources into their diet. On the other hand, there have been increases in meat intake among individuals whose socioeconomic situation has improved, since meat has become much more attractive than fruits and vegetables because of its reduced price.^27^ Meat intake is linked to anthropological and symbolic factors regarding the status of eating meat and this also explains why income is an associated factor.^28^^,^^29^

The association between soft drinks and meat intake can also be explained by the change in cost. It also relates to urbanization and the nutritional transition, since the intake of sugar-sweetened beverages has increased with the reduction in cost of sugar as a commodity and the popularization of these beverages in the Western diet.^27^

The lower meat intake among individuals with two or more chronic diseases and among those with three or more health complaints can be discussed based on the results from Barreto and Figueiredo, who found that the intake of meat with visible fat was inversely associated with the presence of one or more chronic diseases among adults of both genders. This was due to the behavioral change that usually occurs after a chronic disease has been diagnosed. The presence of chronic diseases increases attendance at healthcare services, where individuals receive information relating to health and nutrition. This is usually accompanied by lifestyle modifications in order to minimize the consequences of a disease.^30^

One of the limitations of the present study arose from the application of a single 24-hour dietary recall, which thus did not allow this study to assess the usual diet and limited the possibility of assessing intraindividual variability. Nevertheless, although only one 24-hour recall was collected, these recalls were conducted on different days of the week, and also included weekends and different months of the year, thereby reducing interindividual variabilities.^31^ The 24-hour dietary recall within ISACAMP 2008/2009 was administered to a representative sample of the population of Campinas and therefore enables estimation of the consumption of meat for the city’s population.

Another limitation of the present study was the self-reporting of information on the presence of chronic diseases that had been diagnosed by a physician, and of height and weight data. Although this constitutes a limitation, Almeida et al.^32^ concluded that such information is consistent, through comparing individuals’ self-reported prevalence of chronic diseases and self-assessment of health with the observed impairment of individuals’ daily activities and the existence of situations of being bedridden.^32^ Concerning the use of self-reported height and weight information, epidemiological surveys commonly use self-reported information,^5^ and such data has been shown to be valid.^33^ A study on a similar population demonstrated good comparability between assessed and reported height and weight information among adults.^34^

In addition, since this was a cross-sectional analysis, it provided a snapshot of the population at a single time and associations based on cause and effect cannot be predicted.

## CONCLUSION

In the population of Campinas, the individuals whose meat intake was higher were male, younger adults, individuals with an intermediate family income, those with daily soft-drink intake and those who presented fewer chronic diseases and health complaints. All of these groups presented high average total meat intake, compared with the findings from Brazilian studies and studies in other countries. Furthermore, red meat was the most prevalent type of meat consumed.

The conclusions of this study demonstrate that there is a need for public health interventions from clinicians and researchers with the aims of providing information on the recommended total meat intake and of counseling patients regarding the health risks of high intake of red and processed meats and the importance of choosing lean meats as part of a healthy diet.
